# Fathers’ Experiences of Caring for a Child with a Chronic Illness: A Systematic Review

**DOI:** 10.3390/children10020197

**Published:** 2023-01-20

**Authors:** Shelley Spurr, Cynthia A. Danford, Karyn J. Roberts, Debbie Sheppard-LeMoine, Fernanda Machado Silva-Rodrigues, Michelle Darezzo Rodrigues Nunes, Leslie Darmofal, Anne L. Ersig, Mandie Foster, Barbara Giambra, Stacee Lerret, Michele Polfuss, Lindsay Smith, Suja Somanadhan

**Affiliations:** 1 College of Nursing, University of Saskatchewan, Saskatoon, SK S7N 2Z4, Canada; 2 Office of Nursing Research and Innovation, Cleveland Clinic, Cleveland, OH 44195, USA; 3 College of Nursing, University of Wisconsin-Milwaukee, Milwaukee, WI 53201, USA; 4 Department of Pediatrics, Feinberg School of Medicine, Northwestern University, Chicago, IL 60208, USA; 5 Faculty of Nursing, University of Windsor, Toldo Heath Education Building, 401 Sunset Avenue, Windsor, ON N9B 3P4, Canada; 6 Faculty of Nursing, Santa Casa de São Paulo School of Medical Sciences, São Paulo 01224-001, Brazil; 7 Department of Maternal-Infant Nursing, Nursing Faculty, Rio de Janeiro State University, Rio de Janeiro 20550-013, Brazil; 8 School of Nursing, College of Allied Health and Nursing, Minnesota State University, Mankato, MN 56001, USA; 9 School of Nursing, University of Wisconsin-Madison, 701 Highland Ave, Madison, WI 53705, USA; 10 School of Clinical Sciences, Auckland University of Technology, 90 Akoranga Drive, Northcote, Auckland 0627, New Zealand; 11 Division of Research in Patient Services, Nursing, Cincinnati Children’s Hospital Medical Centre, 3333 Burnet Avenue, Cincinnati, OH 45229, USA; 12 College of Nursing, University of Cincinnati, Cincinnati, OH 45221, USA; 13 Medical College of Wisconsin, 9000 West Wisconsin Avenue, MS B610, Milwaukee, WI 53226, USA; 14 Department of Nursing Research and Evidence-Based Practice, Children’s Wisconsin, 8915 W. Connell Ct, Milwaukee, WI 53226, USA; 15 School of Nursing, University of Tasmania, Launceston, TAS 7250, Australia; 16 UCD School of Nursing, Midwifery and Health Systems, University College Dublin, D04 V1W8 Belfield, Ireland

**Keywords:** fathers, involvement, experiences, chronic illness, systematic review

## Abstract

The prevalence of children living with chronic health conditions is increasing worldwide and can disrupt family roles, relationships, function, and parental involvement in family caregiving. The purpose of this systematic review was to explore fathers’ experiences and involvement in caring for a child with a chronic condition. Systematic searches using seven databases were conducted. Study criteria included (1) peer-reviewed original research in English, Spanish, French, or Portuguese, (2) children less than 19 years of age with a chronic condition, (3) fathers (biological or guardian) as direct informants, and (4) outcomes addressing fathers’ experience, perceptions, and/or involvement in the child’s care. Data were synthesized from ten articles reflecting eight separate studies that utilized quantitative designs. Three areas of focus were identified: *Family Functioning, Father’s Psychological Health*, and *Need for Support.* Data suggested increased involvement from the father in caring for their child with a chronic condition was associated with improved family functioning, increased anxiety and distress, decreased self-esteem, and increased need for support. This review revealed a paucity of data regarding fathers’ experiences and involvement when caring for a child with a chronic condition, with that available primarily from developed countries. Rigorous empirical studies are needed to deepen understanding of how fathers are involved in the care of their child with a chronic condition.

## 1. Introduction

The number of children living with chronic health conditions is increasing worldwide [1]. A chronic condition can be broadly defined as lasting one year or more and requiring ongoing medical care, management of symptoms, lifestyle changes, and limits to the child’s activities of daily living [2,3]. Chronic conditions include physical, psychological, or developmental diseases, and affect several aspects of the child’s life resulting in disruption of daily routines and physical, emotional, and developmental challenges that can strain family relationships and finances [4,5,6]. The prevalence of chronic conditions in childhood is difficult to establish across continents, despite data indicating that approximately 10% of children have a chronic condition [7]. For example, in the United States of America (USA) 10 to 20 million children live with a chronic condition [8], in Canada approximately 500,000 children 15 years or younger have a chronic physical or mental illness [9], in Brazil 9 to 11% of children and adolescents have a chronic disease [10], and in Australia 43% of children have at least one long-term condition [11]. Advances in medical care have resulted in improved treatment of previously disabling diseases, allowing children to live with a severe health condition for months or years, but demands continuous engagement of healthcare systems and families [12].

Having a child with a chronic illness disrupts how families function and relate to one another, and may cause changes in roles, expectations, and responsibilities for daily activities [13]. The disruption of “normal” in family roles and routines, frequent hospitalizations, and daily condition management is stressful [13,14,15], affects the whole family, and requires families to adapt [16] and build resilience [17]. In addition, families require knowledge and preparation to care for their child with a chronic condition. However, the responsibility for caring is often centered on a single person, leading to caregiver overload [18,19]. Mothers are traditionally the primary caregivers for a child with a chronic condition, yet including other family members, particularly fathers, in the care is vital as there is high risk of caregiver burden for a mother who is caring for a child with a complex medical regimen [20,21]. Intentionally or unintentionally leaving fathers out of care can cause frustration and affect their parental identity [22].

Due to recent socio-cultural changes, fathers have become increasingly involved in child-rearing activities, providing more time, care, and emotional support to their children [23]. An increase in fathers’ involvement in advocating for their children’s medical needs has been reported [22] as well as fathers’ participation in caring for a child with a chronic condition [24]. Specifically, a recent review of evidence related to fathering indicated a leveling off of fathers’ time spent providing childcare related to increased involvement, numbers of stay-at-home fathers, and increasing number of states exploring joint physical child custody [24]. However, understanding how fathers are involved in the care of a child with a chronic condition continues to evolve. Thus, the overall aim of this systematic review was to describe and synthesize the literature related to fathers’ experiences when involved in caring for a child with a chronic condition. Our research question was as follows: What are the experiences of fathers who are caring for a child with a chronic condition and how do they engage in family caregiving? This work emerged from an international collaboration among family nurses representing five countries.

## 2. Materials and Methods

### 2.1. Design and Search Strategy

A systematic review was conducted using guidelines from the Johanna Briggs Institute (JBI) for systematic reviews [25]. A systematic search was conducted in November-December 2020 for peer-reviewed manuscripts in the following databases: CINAHL, Embase, ProQuest, PsychINFO, PubMed, Scopus, and Web of Science. No date limits were applied to the search. Search terms reflected our conceptual framework and directed our search strategy. Specifically, the research team identified five concepts to guide the search and search terms to answer our research question: (1) fathers OR father OR paternal, (2) infant, newborn OR neonate OR children OR child OR adolescents, (3) chronic illness OR chronic disease, (4) involvement OR participation OR engagement, and (5) perception OR attitude OR experience. Concept four reflected fathers’ engagement in caregiving and concept five reflected the fathers’ experience as expressed in our research question. An updated search with date limits of 2020–2022 was conducted in January 2022 to ensure no articles were missed. This search found no additional articles.

### 2.2. Study Selection

**Figure 1:**
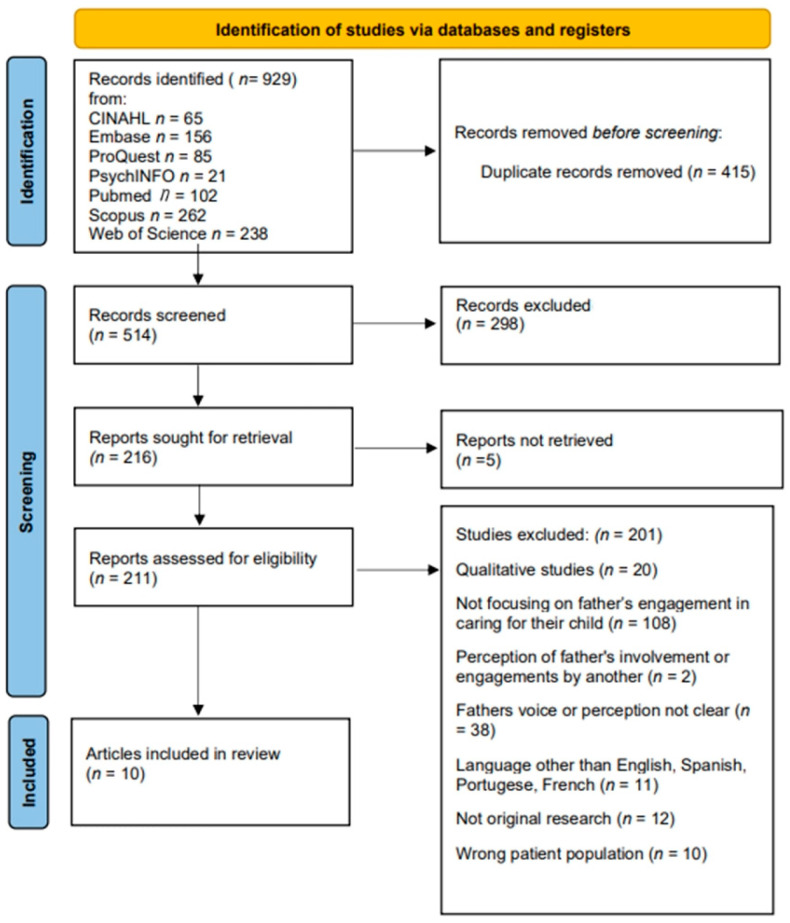
Prisma flow diagram.

A total of 929 individual articles were identified. After removing duplicates, the team of 14 family nurse researchers reviewed 514 individual articles. PRISMA guidelines [26] were followed to identify, screen, and select articles for further review in three stages: titles and citations, abstracts, and full text or article (Figure 1). Three to four members of the research team independently evaluated articles at each stage. Articles were included if records were peer-reviewed original research studies in English, Spanish, French, or Portuguese, included children less than 19 years of age with a chronic condition, and reported fathers’ (biological or legal guardian) experience, perceptions, and/or involvement in the child’s care in the results or outcomes. Articles were excluded if they were not original research (i.e., review article, commentary, etc.), in a language other than English, Spanish, French, or Portuguese, the patient population was older than 19 years of age, the father’s perception, engagement, or voice was not reflected (i.e., someone other than the father was reporting on the father’s engagement or role), or no full text was available. Thirty articles were identified as meeting the inclusion criteria; however, through team discussions, the decision was made to conduct two separate analyses based on the research design. Twenty records employed qualitative designs, while the remaining ten employed quantitative designs. This review and analysis reports on the ten articles utilizing quantitative designs and reflects eight separate studies.

### 2.3. Quality Assessment

All articles were independently evaluated by at least two research team members for methodological quality using the JBI critical appraisal tools for cross sectional (*n* = 9) and cohort studies (*n* = 1) (Table 1 and Table 2). Overall, the quality of the studies was high, demonstrating rigor in study design and methodology.

### 2.4. Review and Extraction

Three to four members of the research team independently extracted data from the included articles and organized the data (with the following fields: author(s), date, design, child age and sex, diagnosis and acuity, country/region of study, sample size of fathers, instruments, and outcomes. The heterogeneity in study characteristics, specifically related to measures and outcomes, precluded a meta-analysis of the data. As such, the next step in the process included three to four members of the research team identifying categories or areas of focus reflected in the outcomes. Identification of areas of focus was guided by the methodology outlined by the Canadian Institutes of Health Research (CIHR) for knowledge synthesis and translation [37]. The narrative involved three stages of synthesis. The first stage involved the organization of articles into logical categories (interventions, ethnic groups, outcomes). The second stage was a within-study analysis and involved a narrative description of the findings of each article. The third stage was a cross-study synthesis that produced an overall summary of the article findings taking account of variations in study quality (variations in populations, interventions, and settings [37]. Through multiple discussions, iterations, and consensus among the research team, data synthesis and application of final areas of focus were completed.

## 3. Results

### 3.1. Study Characteristics

The studies were primarily conducted in developed countries, including the USA [29,30,34,35], Israel [32], Germany [36], Sweden, Australia [27,28], and Norway [31]. One study involved multiple countries, namely the USA, India, Canada, Italy, United Kingdom, and Venezuela [34].

Most of the studies (*n* = 7) were cross-sectional [28,29,30,31,32,33,34] and one was a four-year longitudinal cohort [36]. Considerable variance was noted in terms of the measures and outcomes across the articles. Six articles [27,28,29,30,34,35] used the Dad’s Active Disease Support Scale (DADS), which is designed to measure fathers’ involvement through an assessment of the mothers’ perception of paternal involvement and fathers’ self-report. The Family Assessment Device (FAD) general functioning subscale provided an overall measure of family functioning and was used in three articles [27,28,30]. These same articles used the Impact on Family Scale (IFS) self-report questionnaire and assessed the impact of the child’s chronic illness on the family. One study used the Depression Anxiety Stress Scale (DASS) self-report scale to assess psychological symptoms experienced over the preceding week [27,28]. The Child Behavior Checklist (CBCL) parent report questionnaire was used in two articles and explored the child’s emotions and behavior and provided an overall measure of psychopathology (higher scores indicate greater psychopathology) [28,33]. Across all articles, many measures were used to evaluate various outcomes. Examples of other measures used included the Beliefs about Medication Questionnaire (BMQ), Family Asthma Management System Scale (FAMSS), Asthma Functioning Severity Scale (AFSS), Pediatric Asthma Caregiver Quality of Life Questionnaire, Dyadic Adjustment Scale (DAS), Asthma Control Test (ACT), Family Burden Scale, Hopkins Symptom Checklist-(HSCL-25), Sociodemographic and Illness-related questionnaire, Social Readjustment Rating Scale (SRRS), Self-esteem and Social Support Scales, Marital Adjustment, Father’s Involvement, Family Environment Scale (FES), Family Interaction Task (FIT), Parental Stress Index (PSI), Pediatric Inventory for Parents (PIP), COPE Inventory, Self-care Inventory (SCI-R), and Pediatric Quality of Life.

### 3.2. Participant Characteristics

The participants involved in the articles varied considerably. Three articles solely focused on the experience of fathers who had a child with a chronic condition [32,34,36]. In another six, the participants included both the mother and father; however, the research team was able to extract data obtained from the fathers to include in the review [27,28,30,31,33,35]. Sample sizes ranged from 20 to 249 fathers, and only two articles had less than 50 participants [27,33]. Significant variation also existed in terms of the age of the children (2 days to 18 years) whose fathers participated in the studies; only three articles included infants less than 1 year of age [27,28,32].

The chronic condition of the child varied across the articles. The most common condition studied was type 1 diabetes [30,31,32,34,35,36], followed by asthma [29,30,32,35]. Other chronic conditions experienced by the children included cystic fibrosis, phenylketonuria (PKU), inflammatory bowel disease, spina bifida, imperforated anus, cancer, nephrological diagnosis, juvenile rheumatoid arthritis, and liver disease and/or transplant. The study characteristics are summarized in Table 3.

### 3.3. Areas of Focus

Although there was diversity in method and participant characteristics in the included articles, each contributed to understanding the experiences of fathers who had a child with a chronic condition and how they were involved in family caregiving for the child. Data that captured fathers’ perspectives within the context of the family were clustered into the following focused areas: *Family Functioning, Father’s Psychological Health*, and *Need for Support*. The results reflect fathers’ self-report on the variables discussed. The mothers’ perspective of the fathers’ involvement was not included. The outcomes from each article are presented in Table 4.

#### 3.3.1. Family Functioning

Interactions and relationships in the context of the family environment helped to determine how well a family functioned when a child had a chronic condition. Family functioning collectively included many factors and dimensions and was addressed either directly when fathers completed the FAD (3 articles) [27,28,30] or indirectly when fathers reported on areas in the family that were affected by their child’s chronic condition (5 articles) [27,31,32,33,36]. Two articles did not address components of family functioning [29,35]. In some cases, fathers with a child with a chronic condition rated general family functioning on the FAD as healthier than families with healthy children or those with other illnesses [27,28], whereas other fathers reported better family functioning if they believed themselves to be involved in their child’s care [30]. Other areas affecting family functioning and highlighting its complexity were reflected in the following subcategories: *Fathers’ Role, Relationships, and Communication, Child Characteristics,* and *Mother as Primary Caregiver.*

##### Fathers’ Role, Relationships, and Communication

Challenges to the fathers’ role and family communication were correlated with fathers’ stress scores [28]. For example, disruption to family roles was significantly correlated with adverse psychological symptoms for fathers (*r* = 0.31, *p* < 0.05), particularly related to stress (r = 0.32, *p* < 0.05). In some cases, when the adolescent had a chronic condition, fathers reported taking a less active role in guiding and supporting their adolescent’s independence during family discussions in contrast to the more active communicative role exhibited by fathers of healthy adolescents [36]. Fathers described having less energy and were less goal-oriented compared to fathers with healthy adolescents, and were more easily able to support their adolescent in non-illness-related activities versus illness management [36]. Fathers’ perception of their own involvement with the child was rated lower when compared to that of the mother [29,33].

Marital relationships as a part of family functioning were associated with fathers’ involvement in family caregiving. Helpfulness, a component of fathers’ involvement, measured by DADS, was found to be significantly associated with family functioning and marital relationships (*p* = 0.05) [30]. In one article, fathers expressed a decrease in marital satisfaction when they experienced a greater number of stressful events (*r* = −0.24, *p* < 0.05) [32]. Some fathers reported coping well amidst the strain on marital and parental relationships [33].

##### Child Characteristics

Family functioning was influenced by child characteristics including sex, age, and degree of child illness. In one article, fathers reported better family relationships with their daughters than with their sons with a chronic condition (*p* = 0.02) [36]. However, fathers engaged their sons more often than their daughters in family communication and responsibilities, asking questions, seeking clarification, and offering ideas for problem resolution [36]. Child age was reported to influence family function when, for example, family function was found to be in a healthy range during infancy [27]. Yet, another article found treatment adherence decreased as child age increased and fathers reported low or moderate helpfulness [30]. Related to degree of illness, fathers reported that a higher impact of illness on the child’s family resulted in greater problems in family role (r = 0.48; *p* < 0.001) [28]; family burden was also high when health concerns were long term [31]. On the other hand, the family environment tended to be more [33] structured as reported by fathers of adolescents with diabetes versus fathers of healthy adolescents (*p* = 0.008) [36].

##### Mother as Primary Caregiver

It was not uncommon for the fathers to report the mother was the primary caregiver of the child with the chronic condition. Generally, fathers reported that they took less responsibility for and perceived themselves to be less involved in managing their child’s illness than the child’s mother [29]. This was also reflected in the results from the DADS [28,29,30]. Some discrepancies also presented in that some fathers reported that the mothers assume they themselves take on more responsibilities than the fathers believe [33] and mothers do not give credit for fathers’ involvement. Other fathers perceived themselves to be less helpful and rated their contributions as less helpful than mothers to family functioning [29].

#### 3.3.2. Fathers’ Psychological Health

Fathers’ psychological health as related to their involvement with their child with a chronic condition presented as one comprehensive area of focus. Reported in a variety of ways, fathers’ psychological health included anxiety [5], emotional distress or stress [27,31,32,34], self-esteem [32], burden [31], coping [34], and concerns about quality of life [29,30,35].

Some fathers who rated themselves as having high anxiety also reported being more involved and helpful with their child [28]. However, it was unclear whether interaction with the child adversely caused anxiety in fathers or if the father’s anxiety positively led to greater engagement with their child with a chronic condition [28].

Fathers’ involvement in family caregiving of their child with a chronic condition positively correlated with stressful life events [32]. Specifically, fathers of children with chronic conditions experienced a greater number of stressful life events (personal, family, and social) and expressed feelings of lower self-esteem more than fathers of healthy children (*F* (1,157) = 8.95, *p* < 0.005) [32]. Fathers reported that night-time care and long-term health concerns increased family burden [31]. For example, night-time care (blood glucose measurements) was significantly associated with burden, and adverse events at night (hypoglycemia) were significantly associated with parental emotional distress, including that of fathers [31].

Teasdale and Limbers [34] also reported that fathers who were more involved with their chronically ill child had higher stress levels and used denial as a coping mechanism. Coping through denial seemed to be an effective strategy motivating fathers to be involved with their child. In those fathers who used denial as a coping strategy, an association was noted between high *general* parenting stress and illness management, which resulted in increased involvement with their child with a chronic condition [34]. Results related to the quality of life of fathers were inconclusive. In some cases, fathers reported that more involvement with their child’s care resulted in lowering their own quality of life and increasing the morbidity of their child [29]. In contrast, some fathers who reported high involvement with the care of their child or adolescent also reported their child had a higher health-related quality of life than that reported for children of fathers who were not involved in their care [30,35].

#### 3.3.3. Need for Support

Support as a need presented in this review in two paradoxical ways that were both influential in the fathers’ ability to be involved in the care of their child with a chronic condition: support provided by fathers and support sought by the fathers. Support provided by fathers was directly referenced in six articles, which used the DADS scale to measure how much fathers supported or were involved in the care of their child with a chronic condition and if their involvement was helpful [27,28,29,30,34,35]. Support sought by the fathers was represented through reference to knowledge about the child’s illness or management of their illness [29] and through interpersonal relationships [32,36].

When reporting on *support provided* for their child with a chronic condition, fathers tended to either self-report less involvement and being less helpful than the mother [29] or they reported no significant difference than the mother in the support they provided their child [28,30], meaning that fathers tend to be under-involved in management of their child’s illness [30]. The lack of support or care they provided may have been related to the lack of support they received, yet this was not directly addressed. However, fathers’ self-reported amount of support provided was positively associated with helpfulness (*p* < 0.0001) [35]. One article revealed an association between high fathers’ self-reported DADS scores with marital and family functioning [30], meaning the more involved they were or the more support they provided the better their marital relationship and family functioning. Another suggested that greater father involvement was associated with social support used for coping [34].

The fathers also reported a need for social support. For example, a significant negative correlation was reported by fathers between stressful life events they experienced and lower social support experienced (r = −0.19, *p* < 0.05) [32]. In addition, a positive correlation was found between self-esteem and increased social support (r = 0.20, *p* < 0.05). Similar results were presented that showed that, over the course of four years, all of the participating fathers sought out social support regardless of their child’s health status (t1 < t2, t3, t4; *p* = 0.04, 0.001, and 0.001, respectively; and, t2 < t3, *p* = 0.032) [36]. However, no significant differences in support were found between those with a child with a chronic condition and those with a healthy child [32,36].

Knowledge was expressed as a form of support needed by fathers. Although only addressed in one article, this tangible, concrete need for support warrants attention. Fathers who had less knowledge on how to assess and manage their child’s asthma symptoms were less responsive to the child [29]. For example, the fathers’ beliefs related to the child’s need for asthma medications were less strong than the mothers’ beliefs. Additionally, the fathers’ ability to collaborate with their child’s provider was generally weak [29]. More knowledge and understanding of the child’s care as a need could contribute to more interaction with and more support provided by fathers to their child with the chronic condition.

## 4. Discussion

This systematic review (SR) explored the experiences of fathers who have a child with a chronic condition and how they were involved in family caregiving. Data were synthesized from ten articles reflecting eight separate studies that utilized quantitative designs. Despite the broad search, most of the data available was from developed countries. This review revealed a paucity of data regarding fathers’ experiences and involvement when caring for a child with a chronic condition. The SR found contradicting evidence among articles and study variables, sometimes indicating significant relationships between variables and at other times not. However, despite the diversity in the study measures and outcomes, participant characteristics, and chronic conditions of the children across the articles, three areas of focus were extracted: *Family Functioning, Father’s Psychological Health*, and *Need for Support.*

### 4.1. Family Functioning

The *Fathers’ Role, Relationships, and Communication* were challenged with the increased stress of caring for a child with a chronic condition, which resulted in mental health problems for some fathers [28]. Often, the family was able to function better with increased help from the father. Yet, when communication was difficult, family functioning was compromised [36]. These findings support the results of previous reports related to the changing roles of parents when adapting to and managing life with a child who has a chronic condition [5,6,13]. Our review highlights the concerns and challenges that fathers identified regarding family roles and communication and the need for research into strategies to improve coping and stress management for fathers who are caring for a child with a chronic condition.

In regard to family roles, fathers perceived the mother as the primary caregiver, which positively influenced family functioning [29,33]. Our review confirms the responsibility for caring for the needs of a child with a chronic condition continues to weigh heavily on the mother [29,33]. Although many fathers reported their own contributions as helpful, they tended to defer much of the child’s care to the mother. In one article, the fathers perceived that the mothers tended to have a greater ability to assess and respond to symptoms and exacerbations of the chronic condition [29]. The uneven distribution of parental responsibility may be due in part to the mothers feeling more comfortable completing the tasks and being more familiar with the child’s routine than the fathers. Additionally, mothers may be reluctant to relinquish the responsibility of care to fathers, based on their maternal instincts and perceived maternal responsibilities [22]. As such, the father’s role may be inadvertently minimized and thus contribute to their perceived lack of ability. Alternately, fathers may be unwilling to take on more responsibility, feel undervalued in their role as a caregiver, or take on more comfortable roles such as working outside the home and managing the finances [29]. Although Yogman et al. [22] argue that paternal involvement has increased over the last decade, this review indicates that paternal involvement continues to be limited and emphasizes the traditional maternal role as the primary caregiver [21,29]. Research is needed to better understand how to enhance the paternal caregiving role and create a better balance between maternal and paternal engagement to support the child and their family.

### 4.2. Father’s Psychological Health

Our review found the psychological health of fathers who are involved in the care of their children with a chronic condition impacted their quality of life and required increased support. Previous research supports the SR findings discussed above and shows parents with a child with a chronic condition have decreased psychological health. For example, one study found fathers’ psychological health (physical and mental health) was significantly lower if a child in the family was diagnosed with a complex pediatric neurological condition [38]. Another study that investigated the impact of having a child with a chronic illness found lower levels of parental psychological health (increased anxiety levels) when the parent(s) had a history of depression [39]. Studies have also investigated the effects of stress and burden on family psychological health when a child has a chronic condition [40,41,42]. In a recent meta-analysis, parents of children with a chronic condition showed small to moderate increased parenting stress [43]. Another study, in which 83% of the participants identified as a woman and 87% of these participants were primary caregivers, reported that depression and negative coping, were risk factors for anxiety, whereas protective factors included an internal locus of control, quality of life, emotional well-being, familism, and positive coping styles [39]. Although these studies report the effects of caring for a chronically ill child, few focus on the psychological health of the father. Targeted interventions designed to enhance problem-solving strategies for fathers should be considered along with approaches to address the fathers’ emotional stress, anxiety, and lower self-esteem.

### 4.3. Need for Support

Fathers of children with a chronic condition need support when they are involved in caregiving. Although few studies focus on fathers’ involvement in the care of a child with a chronic condition, our SR findings found that fathers were involved and supportive in such care [27,28,29,30,34,35]. However, the review identified that some fathers did not have sufficient knowledge or support to care for the complex medical needs of their child [29] and reported increased stress and less social support [32]. These findings are similar to a recent study that found parents experienced challenges as caregivers and perceived a lack of support, which often led to role strain and conflict [44]. Another review study confirmed the need for further support and education for parents with a child diagnosed with cancer [45]. These two studies were similar in that there was a need for further education and support; however, the focus remained on the entire family. Our review evaluated the experience of the fathers and found that targeted strategies to increase social support for fathers both inside and outside their families are needed, along with accessible educational interventions regarding caregiving, disease progression, and daily management. Creative solutions should consider utilizing user-friendly and family-informed technology.

## 5. Strengths and Limitations

This systematic review has several strengths. It is the first known review to address fathers’ involvement in caregiving in families with children with a chronic condition presenting data from fathers as the primary informants. This review was conducted by researchers from five countries, expanding our search beyond studies written in English to include Spanish, Portuguese, and French. This review also has limitations that require consideration. Most of the articles reviewed featured studies that were cross-sectional or correlational in nature, sometimes making it difficult to determine the direction of the relationship and highlighting the lack of studies supporting causal relationships. Our inclusion criteria were strict resulting in a smaller number of included articles. It was sometimes difficult to extrapolate data directly from fathers in articles reporting data from both mothers and fathers. To prevent misinterpretation, data in question were not reported and some nuances from the fathers may have been lost. The participant study characteristics such as chronic conditions and treatments varied significantly. Additionally, the focus of this review was on quantitative methods and measures focusing on fathers’ self-report. Data from qualitative studies are necessary to broaden understanding of fathers’ roles. Finally, although several articles used the same measures, we acknowledge that inconsistencies may exist related to how variables and concepts are defined and relationships interpreted across articles, introducing variability in the findings.

## 6. Conclusions

Caring for a child with a chronic condition is often associated with disruptions in family function and changes in roles, expectations, and responsibilities in family caregiving. This SR explored fathers’ experiences and involvement in caring for a child with a chronic condition. The results highlighted the challenge of deeply understanding fathers’ experiences due to differences in measures, varying outcomes, and the studies being conducted primarily in developed countries. However, three focus areas were identified from the analysis, suggesting increased involvement from the father was associated with improved family functioning but decreased psychological health of the father (increased anxiety and distress, decreased self-esteem); moreover, fathers need more support when caring for a child with a chronic condition. Future studies should include empirical research with experimental and quasi-experimental designs that are focused on exploring what is needed to increase family functioning, psychological health, and support for fathers’ who are caring for their child with a chronic condition. Generating this knowledge may aid in the development of measures and targeted interventions designed to lead to improved outcomes for families caring for a child with a chronic condition.

## Figures and Tables

**Table 1:** Critical appraisal of cross-sectional studies.

JBI Checklist Questions	Bowden et al., 2017 [27]	Bowden et al., 2015 [28]	Friedman et al., 2015 [29]	Gavin and Wysocki, 2006 [30]	Haugstvedt et al., 2011 [31]	Katz and Krulik, 1999 [32]	Ojmyr-Joelsson et al., 2009 [33]	Teasdale and Limbers, 2018 [34]	Wysocki and Gavin, 2006 [35]
Were the criteria for inclusion in the sample clearly defined?	Yes	Yes	Yes	Yes	Yes	Yes	Yes	Yes	Yes
2. Were the study subjects and the setting described in detail?	Yes	Yes	Yes	Yes	Yes	Yes	No	Yes	Yes
3. Was the exposure measured in a valid and reliable way?	Yes	Yes	Yes	Yes	Yes	Yes	Yes	Yes	Yes
4. Were objective, standard criteria used for measurement of the condition?	Yes	Yes	Yes	Yes	Yes	Yes	Yes	Yes	Yes
5. Were confounding factors identified?	NA	NA	NA	NA	NA	NA	NA	NA	NA
6. Were strategies to deal with confounding factors stated?	NA	NA	NA	NA	NA	NA	NA	NA	NA
7. Were the outcomes measured in a valid and reliable way?	Yes	Yes	Yes	Yes	Yes	Yes	Yes	Yes	Yes
8. Was appropriate statistical analysis used?	Yes	Yes	Yes	Yes	Yes	Yes	Yes	Yes	Yes

**Table 2:** Critical appraisal of cohort studies.

JBI Checklist Questions	Seiffge-Krenke, 2002 [36]
Were the two groups similar and recruited from the same population?	No
2. Were the exposures measured similarly to assign people to both exposed and unexposed groups?	Yes
3. Was the exposure measured in a valid and reliable way?	Yes
4. Were confounding factors identified?	Yes
5. Were strategies to deal with confounding factors stated?	Yes
6. Were the groups/participants free of the outcome at the start of the study (or at the moment of exposure)?	Yes
7. Were the outcomes measured in a valid and reliable way?	Yes
8. Was the follow up time reported and sufficient to be long enough for outcomes to occur?	Yes
9. Was follow up complete and, if not, were the reasons to loss to follow up described and explored?	Yes
10. Were strategies to address incomplete follow up utilized?	Yes
11. Was appropriate statistical analysis used?	Yes

**Table 3:** Characteristics of the studies included in this review.

Author(s), Date	Design	Child age and Sex	Diagnosis + Acuity	Country/Region and Community (rural, urban, etc.)	Sample Size of Fathers	Instruments (e.g., DADS)
1. Bowden et al. (2017) [27]	Cross- Sectional design	2–700 days; 15 male, 27 female	Infants recently diagnosed with serious liver disease; biliary atresia most common	Australia	42 families, 37 at follow-up	Family Assessment Device (FAD), Impact on Family Scale (IFS), Depression Anxiety Stress Scale (DASS), Dads’ Active Disease Support scale (DADS), Child Behavior Checklist (CBCL)
2. Bowden et al. (2015) [28]	Cross-sectional design	Infants <2 years (2–700 days); 15 male, 27 female	Serious liver disease (liver disease that may require transplantation in the future)	Four metropolitan children’s hospitals in Australia; Sydney (2), Brisbane (1), Melbourne (1)	42 two-parent families	FAD, IFS, DASS, DADS
3. Friedman et al. (2015) [29]	Cross-sectional design	5–9 years; nonspecific sex	Physician-diagnosed asthma requiring daily controller medication	USA	63 dads, 63 moms	DADS, Beliefs about Medication Questionnaire (BMQ), Family Asthma Management System Scale (FAMSS), TrackCap, Smartinhaler, Asthma Functioning Severity Scale (AFSS), Asthma Control Test (ACT), Pediatric Asthma Caregiver QOL Questionnaire
4. Gavin and Wysocki (2006) [30]	Cross- sectional design	2–18 years; 94 male, 96 female	6 chronic illnesses: asthma (at least mild persistent in sensitivity), cystic fibrosis, type 1 diabetes, phenylketonuria (PKU), inflammatory bowel disease, spina bifida	USA	*n* = 190 hetero-sexual couples	Demographics, DADS, FAD, IFS, Dyadic Adjustment Scale (DAS), Only Maternal perception—Parenting Stress Index brief form, Brief symptom Inventory (BSI)-not included in SR
5. Haugstvedt et al. (2011) [31]	Cross-sectional design	Mean age 10.6 y (SD 3.6, range 1.6–15.9), mean age at diagnosis 6.7 y (SD 3.5, range 1.1–14.3), mean duration of diabetes 3.9 y (SD 2.9, range 0.3–14.2)	Type 1 diabetes	Norway, Hordaland County	97 fathers	The fathers completed the Hopkins Symptom Checklist-25 items (HSCL-25), measuring emotional distress, and the Family Burden Scale, which includes five questions measuring perceived family burden related to the child’s diabetes. Information collected from the fathers included routines for blood glucose measurements, hypoglycemic events, and comorbid somatic diseases. These included the frequency of perceived problematic hypoglycemic episodes during the past year, experience with nocturnal hypoglycemia at least once, and experience with hypoglycemia with unconsciousness at least once and the number of blood glucose measurements per day.
6. Katz and Krulik (1999) [32]	Cross-sectional design	6 months-7 years; chronic illness 44 male, 36 female; healthy 40 male, 40 female	Diagnosed as suffering from a chronic illness (≥6 months), receiving daily medical treatment at home (cancer, nephrological diseases, diabetes, asthma, and juvenile rheumatoid arthritis)	Israel	160 dads (80 of children with chronic illness; 80 of “healthy children”)	Sociodemographic and illness-related questionnaire, shortened version of the Social Readjustment Rating Scale (SRRS), Self-esteem scale, Social Support Questionnaire, Marital Adjustment Test, Father’s involvement in the care of the child
7. Öjmyr-Joelsson et al. (2009) [33]	Cross-sectional design	8–13 years; 9 male, 16 female	Imperforate anus	Stockholm, Sweden	20 dads, 25 moms	Study-specific questionnaire, Swedish version of the CBCL
8. Seiffge-Krenke (2002) [36]	Cohort study design	Mean age 13.9 y; diabetic 47 female, 53 male; nondiabetic 56 female, 44 male	Diabetes	Germany	133 dads [75 fathers of healthy FHA adolescents + 58 fathers of diabetics FDA adolescents]. Drop-out rates were low with FDA 17% and FHA at 13%.	F-Copes, Family Environment Scale (FES), Family Interaction Task (FIT), transcripts of family conversations
9. Teasdale and LImbers (2018) [34]	Cross-sectional design	2–10 years, nonspecific sex	Type 1 diabetes	International (USA, India, Canada, Italy, UK, Venezuela, and other countries with only 1 or 2 participants)	249 dads	Parenting Stress Index (PSI), Pediatric Inventory for Parents (PIP), DADS, COPE Inventory, Self-Care Inventory (SCI-R)
10. Wysocki and Gavin (2006) [35]	Cross-sectional study	2–18 years; 94 male, 96 female	6 conditions: asthma (at least mild persistent in sensitivity), cystic fibrosis, type 1 diabetes, PKU, inflammatory bowel disease, spina bifida	USA	190 hetero-sexual couples	Demographics, DADS, disease-specific structured interview to assess medical treatment adherence (based off Diabetes Self-Management Profile), PedsQOL, subjective health status index from child’s primary healthcare provider, healthcare utilization

**Table 4:** Outcomes of the studies included in this review.

Author/s	Outcomes
1. Bowden et al. (2017) [27]	- Fathers did not report elevated levels of psychological symptoms at either time point - Mothers and fathers reported family functioning in the healthy range when compared to both the published healthy/unhealthy cutoff scores - Mothers’ ratings of impact of illness on the family comparable with published research; fathers’ ratings significantly lower at both time points vs. findings from other published research - Did not find an effect of paternal engagement on parent psychological symptoms, family functioning, or impact of the illness on the family - Although fathers’ DADS amount scores were a significant predictor of fathers’ FAD scores at Time 2, mean FAD scores were in the healthy range - Parents differed in the regression model in that, for fathers, but not mothers, lower socioeconomic status (SES) was a predictor of poorer infant emotional and behavioral outcome
2. Bowden et al. (2015) [28]	- Significant correlations between parent psychological symptoms and family functioning, particularly in relation to role disruption, which was also associated with higher perceived impact of the infant’s illness on the family - Severity of illness associated with fathers’ reports of the impact of the illness and not with family functioning - Significant correlations between parent symptoms and family functioning; particularly for fathers, family role disruption and communication were the only areas of family functioning associated with stress symptoms; role adjustments seem to be the biggest issue - Fathers’ engagement in the medical care of chronically ill child was protective; less depression in mothers who rated fathers as more helpful - Fathers who rated themselves as more engaged in the care of the infant also reported greater anxiety - Mothers’ and fathers’ depression and anxiety scores were correlated - SES significantly predicted fathers’ reports of problems in family functioning (FAD general functioning subscale) and mothers’ reports of the helpfulness of fathers’ engagement in the care of the child; negative association with family functioning (lower SES = more problems in family functioning), positive association with fathers’ engagement (higher SES = greater helpfulness of fathers’ engagement) - Fathers’ IFS scores significantly lower than mothers’ scores; fathers’ total DASS mean scores were lower than mothers’ scores
3. Friedman et al. (2015) [29]	- Mothers found to be more involved in asthma management tasks than fathers - Significant discrepancies between maternal and paternal helpfulness; parental perceptions of helpfulness of paternal involvement not significantly correlated - Parental perceptions of amount of involvement significantly correlated - Maternal and paternal beliefs about necessity of asthma medication and concerns about potential consequences of medication correlated - Fathers scored lower than mothers on measures of asthma knowledge and collaborative relationship with asthma care provider - While fathers are involved in daily asthma management tasks, and that involvement is viewed as helpful to family coping, fathers may not be as knowledgeable or as skilled as mothers
4. Gavin and Wysocki (2006) [30]	- For fathers (Table III), significant effects were also obtained for DADS amount and helpfulness (*p* = 0.003 and *p* = 0.001, respectively). - Higher fathers’ DADS scores were associated with more favorable family functioning - For the IFS scale, no significant effects were found
5. Haugstvedt et al. (2011) [31]	- Fathers reported the greatest family burden related to long-term health concerns; 5% of the fathers scored ≥1.75 on the HSCL-25 - Night-time blood glucose measurements were significantly associated with perceived parental (father) burden, and experiences of nocturnal hypoglycemia were significantly associated with parental (father) emotional distress - Fathers’ perceived family burden scores were not significantly correlated with emotional distress
6. Katz and Krulik (1999) [32]	- Fathers of children with chronic illness are fairly similar to fathers of healthy children in their functioning within the family context; main differences are stressful life events and self-esteem - Fathers of children with chronic illness experienced a greater number of stressful personal events and stressful family and social life events vs. fathers of healthy children and lower self-esteem vs. fathers of healthy children - Significant negative correlation found between life events and self-esteem for fathers of healthy children vs. no significant relationship for fathers of children with chronic illness - The greater the number of stressful life events, the lower the social support reported by fathers of children with a chronic illness - Positive correlations between self-esteem and social support for fathers with a child with chronic illness - Significant negative correlations between life events and marital satisfaction; less marital satisfaction when greater number of stressful life events experienced - Significant positive correlations between social support and self-esteem and marital satisfaction - Involvement in the care of the child correlated positively with stressful life events for fathers of children with chronic illness - No significant differences between the fathers in the research and control groups with regard to the influence of the predictors on marital satisfaction and father’s involvement in the care of the child - The greater the social support and the fewer the number of stressful life events, the higher the marital satisfaction expressed by the fathers
7. Öjmyr-Joelsson et al. (2009) [33]	- Significant differences were found between the care provided by the mothers and the care provided by the fathers; fathers may have taken more responsibility when the child was diagnosed but, as the child got older, they gradually conducted fewer tasks required for care of the specific condition - Significant differences between parental opinions on how much responsibility each parent had taken for the care needed for their child’s condition; mothers appeared to be the main caregiver and took the bulk of responsibility for the care needed for their child’s condition - Large difference between the mothers’ and fathers’ reports of the extent of responsibility they took for the specific care of the child’s condition; tendency for mothers to report they undertake more responsibility for the care needed for their child’s condition than the fathers considered the mothers did
8. Seiffge-Krenke (2002) [36]	- Over the course of 4 years, all of the fathers increasingly sought social support among friends and relatives - Diabetic adolescents’ fathers assumed a less active role in encouraging adolescent’s individuation by supporting the adolescent’s independence in family discussions - All fathers showed significant increases in two scales of the F-Copes that pertained to the seeking of social support and the mobilization of the family to obtain help in cases of family problems - Diabetic adolescents’ fathers’ contributions to the conversation were few in number, markedly vague, and generalized; they made significantly fewer suggestions and articulated fewer opinions compared to healthy adolescents’ fathers - Healthy adolescents’ fathers exhibited a very active role in the family communication; they dealt more actively with their child’s contributions and ideas while at the same time bringing in their own contributions - Diabetic adolescent fathers were more passive; it was difficult for them to promote their child’s independence - Only diabetic adolescents’ fathers used the reframing of problems more often over time - Healthy adolescents’ fathers reported higher scores for personal growth than diabetic adolescents’ fathers did at all times - Diabetic adolescents’ fathers perceived the family climate to be more structured than healthy adolescents’ fathers
9. Teasdale and Limbers (2018) [34]	- More frequent pediatric parenting stress was associated with more use of denial as a coping strategy; greater difficulty with pediatric parenting stress was associated with more use of instrumental social support as a coping strategy - More DADS involvement was associated with greater use of instrumental social support as a coping strategy; greater use of instrumental social support as a coping strategy was associated with SCI-R better adherence to the child diabetes treatment regimen - The interaction between use of denial as a coping mechanism and DADS involvement was significantly correlated with the PSI Child Domain; the positive effect of involvement in T1D management on parenting stress was stronger for fathers who reported greater use of denial as a coping mechanism - Positive association between involvement in T1D management and general parenting stress was stronger for fathers who reported using more denial coping strategies; fathers who reported greater use of denial as a coping mechanism were more likely to experience general parenting stress with increased involvement in their child’s T1D management - Fathers’ who reported greater use of denial as a coping mechanism were more likely to report experiencing general parenting stress; with increased involvement in their child’s T1D management, they were not more likely to report experiencing greater pediatric parenting stress - The interaction terms between use of social support as a coping mechanism and DADS involvement were not significantly correlated with any of the parenting stress measures; while social support as a coping mechanism did not influence the relationship between fathers’ involvement with T1D management and either general or pediatric parenting stress, the use of social support was associated with better diabetes management behaviors when examining Pearson correlations.
10. Wysocki and Gavin (2006) [35]	- The DADS scores for amount and helpfulness were highly corelated for fathers - With respect to DADS scores for treatment adherence, neither the main effect for DADS helpfulness nor the interaction of DADS helpfulness with age were significant for fathers; however, a significant age × DADS tertile interaction effect was obtained for DADS amount - Treatment adherence decreased with increased age of child for those with low or moderate “helpfulness” levels from DADS scores but no decrease in treatment adherence was noted for high helpfulness DADS scores - Peds QOL scores did not differ significantly among youth below 14 years of age as a function of paternal involvement; however, among adolescents ≥14 years of age, PedsQOL scores were significantly higher among those in the high tertile for DADS amount scores - Poorer health status (subjective health status and frequency of ER visits) was associated with increased age of the child; DADS amount and helpfulness were not related to health outcomes so the deterioration in health status as age increased was not influenced by paternal involvement

## Data Availability

Data is contained within the article.

## References

[1] Strickland B.B., Jones J.R., Newacheck P.W., Bethell C.D., Blumberg S.J., Kogan M.D. (2015). Assessing systems quality in a changing health care environment: The 2009-10 national survey of children with special health care needs. Matern. Child Health J..

[2] Stein R.E., Bauman L.J., Westbrook L.E., Coupey S., Ireys H.T. (1993). Framework for identifying children who have chronic conditions: The case for a new definition. J. Pediatr..

[3] Centers for Disease Control and Prevention (2022). About Chronic Disease.

[4] Mendes E.V. (2018). Interview: The chronic conditions approach by the Unified Health System. Cien. Saude Colet..

[5] Beacham B.L., Deatrick J.A. (2015). Children with Chronic Conditions: Perspectives on Condition Management. J. Pediatr. Nurs..

[6] Duarte D., Silva K., Tavares T., Jamal C., Silva P., Rosângela de Sena R. (2015). Care of children with a chronic condition in primary care: Challenges to the healthcare model. Texto Contexto Enferm..

[7] Silva N., Pereira M., Otto C., Ravens-Sieberer U., Canavarro M.C., Bullinger M. (2019). Do 8- to 18-year-old children/adolescents with chronic physical health conditions have worse health-related quality of life than their healthy peers? a meta-analysis of studies using the KIDSCREEN questionnaires. Qual. Life Res..

[8] Barthel D., Ravens-Sieberer U., Nolte S., Thyen U., Klein M., Walter O., Meyrose A.-K., Rose M., Otto C. (2018). Predictors of health-related quality of life in chronically ill children and adolescents over time. J. Psychosom. Res..

[9] Canadian Institute of Child Health (CICH) (2018). The Health of Canada’s Children and Youth: A CICH Profile.

[10] Brazilian Institute of Geography and Statistics (IBGE) (2010). An Overview of Health in Brazil: Access and Use of Services, Health Conditions and Risk Factors and Health Protection: 2008.

[11] Australian Bureau of Statistics (ABS) (2019). Microdata: National Health Survey 2017–2018. https://www.abs.gov.au/statistics/microdata-tablebuilder/available-microdata-tablebuilder/national-health-survey#data-item-lists.

[12] Cipolletta S., Marchesin V., Benini F. (2015). Family Functioning as a Constituent Aspect of a Child’s Chronic Illness. J. Pediatr. Nurs..

[13] Smith J., Cheater F., Bekker H. (2015). Parents’ experiences of living with a child with hydrocephalus: A cross-sectional interview-based study. Health Expect..

[14] Tavares K.O., Carvalho M.D., Pelloso S.M. (2010). What being a mother of a child with cystic fibrosis is like. Rev. Gaucha. Enferm..

[15] Fairfax A., Brehaut J., Colman I., Sikora L., Kazakova A., Chakraborty P., Potter B.K., Canadian Inherited Metabolic Diseases Research Network, in collaboration with the Canadian Inherited Metabolic Diseases Research Network (2019). A systematic review of the association between coping strategies and quality of life among caregivers of children with chronic illness and/or disability. BMC Pediatr..

[16] van der Lee J.H., Mokkink L.B., Grootenhuis M.A., Heymans H.S., Offringa M. (2007). Definitions and measurement of chronic health conditions in childhood: A systematic review. JAMA.

[17] Harrist A.W., Henry C.S., Liu C., Morris A.S. (2019). APA Handbook of Contemporary FAMILY psychology: Foundations, Methods, and Contemporary Issues across the Lifespan American Psychological Association.

[18] Marofi M., Bahrami N., Pahlavanzadeh S. (2019). Effect of a supportive training program on anxiety in children with chronic kidney problems and their mothers’ caregiver Burden. Iran. J. Nurs. Midwifery Res..

[19] Salvador M.D.S., Gomes G.C., De Oliveira P.K., Busanello J., Xavier D.M. (2015). Strategies of Families in the Care of Children with Chronic Diseases. Texto Context.-Enferm..

[20] Javalkar K., Rak E., Phillips A., Haberman C., Ferris M., Van Tilburg M. (2017). Predictors of Caregiver Burden among Mothers of Children with Chronic Conditions. Children.

[21] Katz L.F., Fladeboe K., King K., Gurtovenko K., Kawamura J., Friedman D., Compas B., Gruhn M., Breiger D., Lengua L. (2018). Trajectories of child and caregiver psychological adjustment in families of children with cancer. Health Psychol..

[22] Yogman M., Garfield C.F., Committee on Psychosocial Aspects of Child and Family Health (2016). Fathers’ Roles in the Care and Development of Their Children: The Role of Pediatricians. Pediatrics.

[23] Schoppe-Sullivan S.J., Fagan J. (2020). The Evolution of Fathering Research in the 21st Century: Persistent Challenges, New Directions. J. Marriage Fam..

[24] Khanna A.K., Prabhakaran A., Patel P., Ganjiwale J.D., Nimbalkar S.M. (2015). Social, Psychological and Financial Burden on Caregivers of Children with Chronic Illness: A Cross-sectional Study. Indian J. Pediatr..

[25] Aromataris E., Munn Z. (2020). Joanna Briggs Institute Manual for Evidence Synthesis. Joanna Briggs Inst. Man. Evid. Synth..

[26] Page M.J., McKenzie J.E., Bossuyt P.M., Boutron I., Hoffmann T.C., Mulrow C.D., Shamseer L., Tetzlaff J.M., Akl E.A., Brennan S.E. (2021). The PRISMA 2020 statement: An updated guideline for reporting systematic reviews. BMJ.

[27] Bowden M.R., Ee L.C., Krishnan U., O’Loughlin E.V., Hardikar W., Carmody D., Hainsworth C., Jermyn V., Lee M.-M., Sawyer J. (2017). Family Impact and Infant Emotional Outcomes Following Diagnosis of Serious Liver Disease or Transplantation in Infancy. J. Craniofacial Surg..

[28] Bowden M.R., Stormon M., Hardikar W., Ee L.C., Krishnan U., Carmody D., Jermyn V., Lee M.M., O’Loughlin E.V., Sawyer J. (2015). Family adjustment and parenting stress when an infant has serious liver disease: The Australian experience. J. Pediatr. Gastroenterol. Nutr..

[29] Friedman D., Masek B., Barreto E., Baer L., Lapey A., Budge E., McQuaid E.L. (2015). Fathers and Asthma Care: Paternal Involvement, Beliefs, and Management Skills. J. Pediatr. Psychol..

[30] Gavin L., Wysocki T. (2006). Associations of Paternal Involvement in Disease Management with Maternal and Family Outcomes in Families with Children with Chronic Illness. J. Pediatr. Psychol..

[31] Haugstvedt A., Wentzel-Larsen T., Rokne B., Graue M. (2011). Perceived family burden and emotional distress: Similarities and differences between mothers and fathers of children with type 1 diabetes in a population-based study. Pediatr. Diabetes.

[32] Katz S., Krulik T. (1999). Fathers of Children with Chronic Illness: Do They Differ from Fathers of Healthy Children?. J. Fam. Nurs..

[33] Öjmyr-Joelsson M., Nisell M., Frenckner B., Rydelius P.-A., Christensson K. (2009). A Gender Perspective on the Extent to which Mothers and Fathers Each Take Responsibility for Care of a Child with High and Intermediate Imperforate Anus. J. Pediatr. Nurs..

[34] Teasdale A., Limbers C. (2018). Avoidant coping moderates the relationship between paternal involvement in the child’s type 1 diabetes (T1D) care and parenting stress. J. Child Health Care.

[35] Wysocki T., Gavin L. (2006). Paternal Involvement in the Management of Pediatric Chronic Diseases: Associations with Adherence, Quality of Life, and Health Status. J. Pediatr. Psychol..

[36] Seiffge-Krenke I. (2002). “Come on, say something, dad!”: Communication and coping in fathers of diabetic adolescents. J. Pediatr. Psychol..

[37] Grimshaw J. A Knowledge Synthesis Chapter. https://cihr-irsc.gc.ca/e/documents/knowledge_synthesis_chapter_e.pdf.

[38] van Nimwegen K.J., Kievit W., van der Wilt G.J., Schieving J.H., Willemsen M.A., Donders A.R., Verhaak C.M., Grutters J.P. (2016). Parental quality of life in complex paediatric neurologic disorders of unknown aetiology. Eur. J. Paediatr. Neurol..

[39] Toledano-Toledano F., Moral de la Rubia J. (2018). Factors associated with anxiety in family caregivers of children with chronic diseases. Biopsychosoc. Med..

[40] Abrams H.R., Leeds H.S., Russell H.V., Hellsten M.B. (2019). Factors Influencing Family Burden in Pediatric Hematology/Oncology Encounters. J. Patient-Cent. Res. Rev..

[41] Picardi A., Gigantesco A., Tarolla E., Stoppioni V., Cerbo R., Cremonte M., Alessandri G., Lega I., Nardocci F. (2018). Parental Burden and its Correlates in Families of Children with Autism Spectrum Disorder: A Multicentre Study with Two Comparison Groups. Clin. Pr. Epidemiol. Ment. Health.

[42] Fitzgerald C., George S., Somerville R., Linnane B., Fitzpatrick P. (2018). Caregiver burden of parents of young children with cystic fibrosis. J. Cyst. Fibros..

[43] Pinquart M. (2018). Parenting stress in caregivers of children with chronic physical condition-A meta-analysis. Stress Health.

[44] Koch A., Kozhumam A.S., Seeler E., Docherty S.L., Brandon D. (2021). Multiple Roles of Parental Caregivers of Children with Complex Life-Threatening Conditions: A Qualitative Descriptive Analysis. J. Pediatr. Nurs..

[45] Williams P.D., Williams K.A., Williams A.R. (2014). Parental caregiving of children with cancer and family impact, economic burden: Nursing perspectives. Issues Compr. Pediatr. Nurs..

